# HHV-6A/B Integration and the Pathogenesis Associated with the Reactivation of Chromosomally Integrated HHV-6A/B

**DOI:** 10.3390/v9070160

**Published:** 2017-06-26

**Authors:** Vanessa Collin, Louis Flamand

**Affiliations:** 1 Division of Infectious and Immune Diseases, CHU de Quebec Research Center-Laval University, Québec, QC G1V 4G2, Canada; Vanessa.Collin.2@ulaval.ca; 2 Department of Microbiology, Infectious Disease and Immunology, Faculty of Medicine, Laval University, Québec, QC G1V 0A6, Canada

**Keywords:** inherited chromosomally-integrated HHV-6A/B, telomere, integration, human herpesvirus 6A/B, reactivation

## Abstract

Unlike other human herpesviruses, human herpesvirus 6A and 6B (HHV-6A/B) infection can lead to integration of the viral genome in human chromosomes. When integration occurs in germinal cells, the integrated HHV-6A/B genome can be transmitted to 50% of descendants. Such individuals, carrying one copy of the HHV-6A/B genome in every cell, are referred to as having inherited chromosomally-integrated HHV-6A/B (iciHHV-6) and represent approximately 1% of the world’s population. Interestingly, HHV-6A/B integrate their genomes in a specific region of the chromosomes known as telomeres. Telomeres are located at chromosomes’ ends and play essential roles in chromosomal stability and the long-term proliferative potential of cells. Considering that the integrated HHV-6A/B genome is mostly intact without any gross rearrangements or deletions, integration is likely used for viral maintenance into host cells. Knowing the roles played by telomeres in cellular homeostasis, viral integration in such structure is not likely to be without consequences. At present, the mechanisms and factors involved in HHV-6A/B integration remain poorly defined. In this review, we detail the potential biological and medical impacts of HHV-6A/B integration as well as the possible chromosomal integration and viral excision processes.

## 1. Introduction

The human herpesvirus family is divided into three subfamilies: these include the betaherpesviruses, among which are the human herpesvirus 6A, 6B, and 7, and the cytomegalovirus (HHV-6A/B, 7 and CMV). The discovery of HHV-6A dates to 1986, while that of HHV-6B was first reported in 1988 [1,2,3]. In 2012, The Committee on Viruses Taxonomy recognized that two distinct HHV-6 existed and named them HHV-6A and HHV-6B [4]. Both viruses share the same architecture, share 90% homology in their nucleic acid sequences and have a tropism for T CD4+ lymphocytes. On the other hand, they exhibit epidemiological, biological and immunological differences [5,6,7,8]. HHV-6B is a ubiquitous virus that infects nearly 90% of the world’s population in the first two years of life and is the etiologic agent of exanthema subitum [9]. Infection by HHV-6B is characterized by fevers, skin rashes, respiratory distress, and epileptic seizures [9,10]. On the other hand, HHV-6A infections are less characterized, presumably because it is acquired later in life and since most of the population has already been infected with HHV-6B, cross reactive immunity likely controls, at least partially, HHV-6A primary infections resulting in subclinical symptoms.

Certain herpesviruses have developed the ability to integrate their genomes into host chromosomes. Among animal herpesviruses, the lymphotropic alphaherpesvirus Marek’s disease virus (MDV) readily integrates its genome in the telomeric regions of chromosomes upon infection of chicken cells (reviewed in [11]). MDV integration, combined with expression of the *Meq* oncogene and *vTR*, a viral telomerase homolog, are associated with lymphoma development in chicken and represent an important agricultural problem causing important economic losses to the industry. Presumably MDV uses integration as a mean to achieve latency. Integration is a very efficient way to evade immune surveillance. By a yet to be fully understood mechanism, the integrated MDV can excise itself from the host genome and return to the lytic phase of its life cycle. 

Among human herpesviruses, HHV-6A/B are somewhat unique in their ability to integrate their viral genomes into the telomeres of the host’s chromosomes. In 1993, Luppi et al. published a case report of three individuals with a chromosomally integrated HHV-6 (ciHHV-6) [12]. The particularity of these individuals resides in the fact that all three had ciHHV-6 in most peripheral blood cells. The hypothesis that ciHHV-6 could be inherited was validated by Daibata et al. [13,14]. Extrapolation from several studies indicate that approximately 1% of the world’s population (75 million people) has inherited chromosomally-integrated HHV-6A/B (iciHHV-6A/B). Depending on the regions sampled, the proportion of iciHHV-6A ranges from 10% to 40% [15,16]. How integration occurs and a better understanding of the impact of integration on cellular homeostasis represent research priorities. In this review, potential impacts of HHV-6A/B integration at the cellular and the host levels and the biology behind HHV-6A/B integration will be discussed.

## 2. Biological and Medical Consequences Associated with HHV-6A/B Integration

One important biological aspect associated with HHV-6A/B integration is that viral integration can occur in germ cells. When fertilization occurs with a gamete containing ciHHV-6/B, this leads to individuals carrying one (or more) integrated HHV-6A/B copy in every somatic cell. Although the integration of a 160 kilobase pair (kbp) viral genome into telomeres might compromise the chromosomal integrity within cells, consequences are poorly understood due to the lack of clear-cut clinical associations. Pellet et al. gathered results from multiple independent studies and reported that iciHHV-6A/B was more prevalent in diseased individuals compared to healthy subjects [17]. The potential impacts of iciHHV-6A/B are listed below.

### 2.1. False Diagnostic of Active HH-6A/B Infection

One important problem associated with iciHHV-6A/B is the false diagnosis of active HHV-6A/B infections. For most herpesviruses, detection of viral DNA in biological samples is associated with ongoing active infection. In a subject with iciHHV-6A/B, considering that all cells carry a copy of HHV-6A/B, the viral load, especially when assaying whole blood, will be high and most often not be associated with active infections. During an active infection, the number of HHV-6A/B copies per milliliter of blood is generally lower than for iciHHV-6+ subjects. Viral loads associated with active HHV-6A/B infections typically vary between 10^3^ and 10^4^ HHV-6A/B copies/mL. In contrast, the iciHHV-6A/B DNA content is approximately 1–2 × 10^6^ copies/mL [18,19]. Thus, when a high viral load is detected, a second assay (e.g., quantitative PCR of HHV-6A/B copies on hair follicles) to confirm or rule out iciHHV-6A/B should be performed. If negative for iciHHV-6A/B, the patient should be treated with antivirals. The fact that some patients are treated with antiviral drugs in the absence of an actual viral infection can be problematic, especially in vulnerable patients such as those that underwent hematopoietic stem cell transplantation. For this reason, it is very important to differentiate between active HHV-6A/B infection and iciHHV-6A/B. 

### 2.2. iciHHV-6 and Immunodeficiency

Endo et al. reported in vivo reactivation of iciHHV-6A in a boy afflicted with severe combined immunodeficiency linked to X (SCID-X). Integrated HHV-6A reactivation led to the production of infectious virions that caused disease [20]. Fortunately, the boy was successfully treated with antivirals and recovered. This is the first study to provide convincing evidence that, under profound immunosuppression, reactivation of iciHHV-6A/B can occur and cause disease.

### 2.3. Disease and Chromosome Associations

Pathologies associated with iciHHV-6A/B are still poorly understood. Although HHV-6A/B integrate in telomeres, the integration can occur in different chromosomes and for this reason, the pathologies associated with this condition are likely to vary greatly. iciHHV-6A/B was observed in chromosomes 9q, 10q, 13q, 17p, 18q, 19q and 22q [13,14,21,22]. For instance, chromosome 17 (C17), which appears to be a hotspot for HHV-6A/B integration, is associated with multiple genetic diseases. C17 codes for genes such as *TP53*, *BRCA1*, and *RDM1* that are responsible for DNA damage responses and the cell cycle arrest during cell damage. Moreover, loss of the short arm of C17, including telomeric regions, is often associated with rare diseases such as Miller–Dieker Syndrome. Hence, knowing that specific chromosomes are prone to mutations, HHV-6A/B’s integration in such chromosomes could be a source for additional chromosomal instability. 

### 2.4. iciHHV-6 and Telomeres Length

Telomeres are the main protectors of chromosomes and it is expected that a viral insertion at this site could compromise their integrity. In sperm cells, the telomere length of the chromosome carrying ciHHV-6A/B is as long if not longer than that of other chromosomes. In contrast, in somatic cells, the chromosome carrying ciHHV-6A/B is often the shortest [21]. Considering that the telomere erosion rate of chromosomes is similar (independent of ciHHV-6A/B), the reason why the telomere of the chromosome with ciHHV-6A/B is often the shortest remains unclear. One possible explanation is that HHV-6A/B integration might alter transcription of subtelomeric genes such as telomere repeat-encoding RNA (TERRA). In fact, it is known that alteration in TERRA transcription can contribute to telomeres shortening [23,24,25]. The long-term consequence of a shortened telomere could cause premature senescence or cell apoptosis when numerous cell divisions are required. 

### 2.5. iciHHV-6 and Heart Disease

Recently, it was reported that iciHHV-6+ individuals have increased risks of developing heart disease than iciHHV-6- individuals. In fact, Gravel et al. carried out a study of 20,000 Quebeckers among which 113 are iciHHV-6+ (57% HHV-6B and 43% HHV-6A). By comparing 50 chronic diseases, the results suggest that iciHHV-6+ individuals are 3.3× more at risk of developing angina pectoris [15]. In 2015, Das et al. published a case report in which a neonate was hospitalized due to acute heart failures (HF) [26]. After carrying out multiple tests in relation to HF, such as viral loads of CVM and EBV, every test came out negative. Interestingly, the only positive test was for the presence of HHV-6 DNA with one copy/cell, indicative of iciHHV-6. Unfortunately, the neonate died after 16 days of hospitalization but this case raises the possibility of linking iciHHV-6 and cardiomyopathy (DCM). Finally, HHV-6 presence was detected in myocardium biopsies of patients with myocarditis. HHV-6 transcripts and viral envelope proteins were detected in the same cells, indicating the presence of an active infection. When treated with ganciclovir, which eliminated the presence of active HHV-6 virus, the patient’s myocarditis greatly improved [27]. Overall, whether the integration itself plays a role in heart disease remains to be clarified but several studies suggest a role for HHV-6 in heart diseases. There is an outgrowth body of literature linking telomere length with heart disease, in which the telomeres are shorter [28,29]. It can be hypothesized that the shortening of telomeres could be associated with susceptibility to heart disease and, by this very fact, the integration of HHV-6 might accelerate the onset of heart disease development. 

### 2.6. Other Associated Impacts of HHV-6A/B Integration

Since iciHHV-6 individuals carry a viral copy in every cell, all cells are susceptible to express, at some point in time, viral antigens. Considering that everyone is immune to HHV-6B, detection of these proteins would cause tissue damage. Such immune attacks occurring over decades may lead to diseases analogous to autoimmune disease. Moreover, when endothelial cells express HHV-6A/B antigens, cell destruction could lead to blood vessel damages that over time develop into chronic inflammation, plaque formation and stenosis of blood vessels. Studies suggesting spontaneous gene expression in cells isolated from chromosomally integrated HHV-6 do exist. Strenger et al. reported that freshly-isolated leukocytes from iciHHV-6+ individuals spontaneously express HHV-6 mRNA and proteins [30]. Several studies have also documented spontaneous or chemically-induced HHV-6 mRNA expression in cells isolated from iciHHV-6+ individuals [13,18,21]. Considering that spontaneous expression of HHV-6 antigens in iciHHV-6+ subjects can occur in every cell (theoretically), iciHHV-6+ individuals are expected to have a higher HHV-6 antigenic burden than iciHHV-6- subjects. In support, recent work by Strenger et al. suggests that iciHHV-6+ individuals have a higher frequency of circulating HHV-6-specific T cell relative to iciHHV-6- subjects [31]. Considering that another group failed to reach similar conclusions [32], more studies, including larger number of iciHHV-6+ subjects analyzed, are needed before firm conclusions can be drawn. Interestingly, recent work by Hill et al. indicates that acute graft versus host disease (aGVHD) grades 2–4 were more frequent when recipients or donors had iciHHV-6 [33]. Whether expression of HHV-6 antigens is a trigger for aGVHD in hematopoietic stem cell transplant recipients warrants investigation.

## 3. HHV-6’s Home, the Telomeres

In vertebrates, each chromosome possesses several kbp of non-coding hexanucleotides (TTAGGG)_n_ that terminates with a single-stranded G rich 3′ overhang of 30–500 nucleotides [34,35]. Telomeres play two major roles in protecting the chromosomes. First, they prevent the loss of genetic information. During DNA replication, the DNA polymerase is unable to copy the end of the lagging strand, which causes end replication problem. Consequently, at each cell division, telomeres are shortened by 50–100 nucleotides. When the number of hexameric repeats falls below 13, cells stop proliferating and undergo senescence or apoptosis [36,37]. Hence, in the absence of telomere elongation mechanisms, each cell has a predetermined number of cell divisions that is influenced by the length of the telomeric repeats. Second, telomeres protect chromosomes against the DNA damage response (DDR), DNA recombination and DNA end joining that causes chromosomes instability [38,39]. Protection against such events is realized when the single-stranded 3′ overhang of telomeres is buried in the double stranded telomeric region to form a telomeric loop (t-loop) [40,41]. This t-loop is made and maintained by the shelterin complex that acts as the guardians of the telomeres.

### 3.1. Telomeres Replication and Transcription

Telomeres are synthesized and elongated in stem cells by the telomerase complex [42]. Telomerase is composed of a multi subunits complex such as telomerase reverse transcriptase (TERT), the telomerase RNA component template (TERC), DKC1, NoP10, Reptin, Pontin and NHP2. The replication of telomeres is made by telomerase TERT that uses TERC as a telomere template to elongate the latter [43,44]. When cells are differentiated, telomerase activity is barely detectable because of the silencing of TERT. In contrast, TERT expression is reactivated in 80–90% of cancer cells [45]. Moreover, it has long been thought that telomeres were transcriptionally silent until the discovery of TERRA non-coding RNA whose transcription is initiated in the sub-telomeric region and extends within the telomeric sequences. TERRA has been shown to stabilize shelterin proteins and promote heterochromatin state by interacting with telomeric repeat binding factors 1 and 2 (TRF1 and TRF2) [46]. In addition, TERRA acts as a long non-coding RNA to regulate telomerase activity [47]. 

### 3.2. Guardians of Telomeres

The shelterin complex is composed of six proteins: TRF1 and TRF2, protection of telomere 1 (POT1), telomere protection protein 1 (TPP1), TRF interacting nuclear protein 2 (TIN2) and repressor activation protein 1 (RAP1) [48]. Heterodimers of TRF1 and TRF2 bind to the double stranded telomeric DNA via their myb domain. TRF1 is involved in telomere replication and prevents blockage of the replication fork. The role of TRF2 is to protect telomeres against the DNA repair mechanisms by inhibiting the Ataxia-telangiectasia-mutated (ATM) pathway. When ATM is not inhibited, the double stranded DNA break (DSDB) repair mechanisms are activated [38,49,50,51]. Ultimately, a loss of TRF2 leads to telomere recombination or end to end chromosomes fusions, and such consequences can lead to cell apoptosis or neoplastic cells [52,53]. The single stranded DNA (ssDNA) of telomeres is bound by POT1, a protein that inhibits the Ataxia-telangiectasia and Rad3 related (ATR) pathway that is responsible for single stranded DNA break (SSDB) damage repair mechanism activation. Moreover, TRF2, together with POT1 also inhibits homologous recombination (HR) [42]. The bond between the rest of the complex and POT1 is made by TPP1, and TIN2, which stabilize the whole complex. Finally, RAP1 binds TRF2, which prevents HR and non-homologous end joining (NHEJ) [52]. Thus, TRF2 is the pivotal protein for maintaining the integrity of telomeres and chromosomes.

## 4. Herpesviruses Infection and Latency

### 4.1. Viral Cycle of Herpesviruses

During herpesvirus infection, a series of sequential events occurs. First, the virus binds to cell surface receptors present on the host cell. Following fusion of viral envelope with the cell membrane, the viral capsid is released in the cytoplasm and degraded, liberating the viral DNA that is transported into the nucleus [54]. Once in the nucleus, the viral DNA circularizes and expresses immediate early genes (IE). Among many functions, the IE proteins activate the expression of the early genes, encoding proteins associated with viral DNA replication. Once DNA replication has occurred, expression of late genes is turned on. For the most part, late genes encode structural proteins required for the assembly of new virions. Once assembled, progeny viruses are released [55,56]. However, not all herpesvirus infections lead to a productive infection and release of infectious virions. In some cases, the virus enters a cell and enters a state of latency. 

### 4.2. Herpesviruses Latency

The classical mode of latency is by maintaining the viral genome as episomes, tethered or not, to the human chromosomes. For example, the EBNA-1 protein of Epstein Barr Virus (EBV) and LANA protein of HHV-8 tether the viral episomes to the host chromatin ensuring that upon cell division, daughter cells will contain viral episomes [57,58]. Generally, infection of permissive cells (such as T lymphocytes) by HHV-6A/B results in a lytic infection with no latent phase detected. In fact, in vitro models of latency where the HHV-6A/B genome is maintained as episomes have yet to be identified or studied in sufficient detail. Using HHV-6-infected primary human macrophages, Kondo et al. reported latent HHV-6 infection, with the possibility of reactivating the virus using phorbol ester stimulation [59]. In latently-infected cells, large viral transcripts initiating upstream and extending beyond the major IE locus were detected [60,61]. Unfortunately, the state (episome, linear, and integrated) of the HHV-6 genomes in these cells was not reported. In contrast to permissive cells, when HHV-6A/B infects semi- or non-permissive cells with little or no DNA replication, the outcome is either an abortive infection or chromosomal integration [62]. The latency-associated HHV-6 proteins are also unknown. Transcripts coding for the HHV-6A/B U94 protein were reported to be expressed in peripheral blood mononuclear cells (PBMCs) of healthy subjects and hypothesized that U94 might play a role in latency [63]. This is supported by the fact that U94 expression represses HHV-6A/B DNA replication and therefore might favor the establishment of latency [64]. U94 is a protein that shares 24% homology with the REP68/78 integrase of the Adeno-associated virus type 2 (AAV-2), a protein responsible for AVV-2 chromosomal integration [65]. It was shown that U94 can bind DNA and complement the activity of REP68/78. For this reason, it was surmised that U94 might play a role in the establishment of latency and/or chromosomal integration [64,66]. Furthermore, because of its activities, it was thought that U94 was implicated in HHV-6A/B integration. However, Wallaschek et al. showed that a U94 deletion mutant of HHV-6A was as proficient as WT HHV-6A at integrating human chromosomes [67]. Hence, more investigation is required to identify HHV-6A/B models of latency, associated proteins and nature of the latent genome. 

## 5. HHV-6A/B Genomes and Their Integrated Forms

### 5.1. Genome

**Figure 1:**
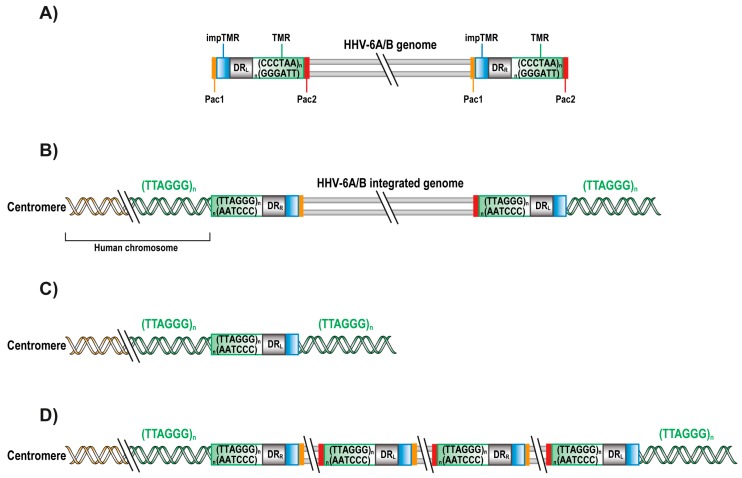
HHV-6A/B genomes and their integrated forms. Schematic representation of human herpesvirus 6A and 6B (HHV-6A/B) genomes and the reported integrated forms. (**A**) The unique region (U) of the 160 kbp HHV-6A/B genomes is flanked by identical direct repeats (DR_L_ and DR_R_) of 8–9 kbp. The DRs possess a *pac1* (yellow) and *pac2* (red) sequences, adjacent to imperfect telomeric repeats impTMR (blue) and TMR (green) sequences, respectively. The genome is not drawn to scale; (**B**) Chromosomally integrated HHV-6A/B (ciHHV-6A/B) genome (with loss of *pac2* in DR_R_ and *pac1* in DR_L_) with elongated telomeres at the DR_L_; (**C**) Single integrated DR_L_ with elongated telomere; (**D**) Integrated HHV-6A/B concatemers. Genomes are not drawn to scale.

During infection, the HHV-6A/B genome is present in three different forms. The first form of HHV-6A/B’s genome is linear, which is present in the virus and the cell upon infection. Once in the nucleus, the viral genome circularizes to form an episome. The episome is used as a template for viral replication during which viral concatemers are formed. HHV-6A/B genome is approximately 160 kbp in length with a unique region (U) containing more than 100 open reading frames (ORFs) and encoding more than 97 proteins expressed sequentially throughout the lytic cycle of the virus [5,68,69]. The extreme end of the viral genome is flanked by two identical directly repeated (DR_L_ and DR_R_) regions of 8–9 kbp. The 5′ end of the DR contains a *pac*1 sequence (56 bp) while the 3′ end contains *pac*2 sequence (80 bp), responsible for the cleavage and packaging of the viral genome. Adjacent to *pac*2 are 15–180 reiterations of TTAGGG telomeric repeats (TMR), identical to human telomeric sequences. Adjacent to *pac*1 are imperfect TMR (impTMR) (Figure 1A) [68,70,71]. 

### 5.2. Integrated HHV-6A/B

For most herpesviruses, integration into host chromosomes is an unusual event. In some cases, it was observed that in the presence of DNA breaks induced by UV radiation, human herpes simplex virus type 1 and 2 (HSV-1 and HSV-2) can integrate into chromosomes. However, the integrated HSV-1 and HSV-2 genome were fragmented and mutated, not able to generate new virions [72]. In contrast to HSV-1 and HSV-2, HHV-6A/B integrated genomes remain largely intact with their ORFs conserved. Analysis of cells with ciHHV-6A/B indicates that these viruses are mostly integrated into telomeres with DR_R_ fused to the chromosome (Figure 1B) [21,73]. In fact, HHV-6-A/B DR_R_ are adjacent to the subtelomeric portion of the human telomere with loss of the *pac*2 sequence at DR_R_ and loss of the *pac*1 sequence at DR_L_. Such a structure is compatible with integration occurring by HR events. In addition, at the DR_L_ end of the integrated genome are impTMR that appear to serve as a template for telomere elongation by the telomerase complex or alternative lengthening mechanisms [74]. In occasional cases, integrated HHV-6A/B consists of a single DR fused to the chromosome, a structure that is compatible with integration occurring by HR events initiated at DR_L_ (Figure 1C). Alternatively, individuals containing multiple contiguous HHV-6A/B copies also exist. Such structure can be explained by integration of a viral concatemer (Figure 1D). By assessing the number of DR present, one can determine the number complete HHV-6A/B’s genomes that is/are integrated.

### 5.3. Excision of Integrated HHV-6

**Figure 2:**
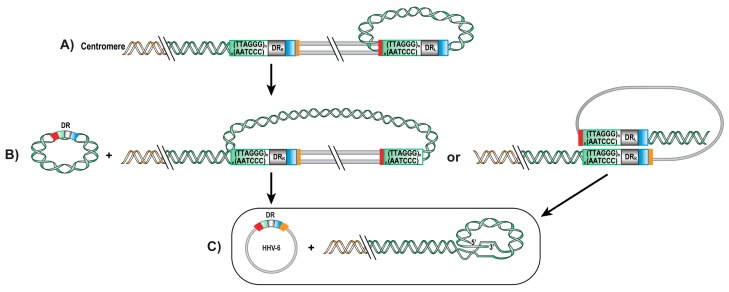
Possible mechanisms of HHV-6A/B genome excision from telomeres. Schematic representation of hypothetic processes of HHV-6A/B genome excision from telomeres. (**A**) Telomeric repeats form a t-loop in the TMR of HHV-6A/B DR_L_, followed by recombination and excision, resulting into a first t-loop excision: a telomeric circle and a chromosomally integrated HHV-6A/B lacking a DR but still possessing TMR sequences. (**B**) A second t-loop formation is made by recombination of the TMR at the end of the genome into HHV-6A/B DR_R_, resulting in a fully excised and circular HHV-6A/B genome containing a single DR with a single *pac1*, *pac2*, impTMR and TMR sequence. (**C**) Invasion of the telomeric repeats into the TMR of the DR_R_, resulting into a HHV-6A/B free chromosome and a full viral genome with a complete DR.

To ensure their long-term maintenance in any given population, integrated viruses must be able to re-initiate a lytic cycle and generate progeny virions. A previous study has reported the presence of an extrachromosomal circular HHV-6 genome with a single DR in cells from an iciHHV-6 subject [21]. This led to the conclusion that the viral genome can be excised from telomeres by one or two t-loop formation and recombination. To explain the presence of a single DR in the excised viral genome, one hypothesis is that excision occurs by a two-step t-loop formation (Figure 2A). At each cell cycle, telomeres are shortened and reform a t-loop to protect the chromosomes. First, the telomere sequence at the end of the DR_L_ could form a t-loop by invasion of the TMR within DR_L_ itself. This t-loop formation would result in a t-loop excision, forming a telomeric circle with a single DR and an intermediate form of the HHV-6A/B integrated chromosome (Figure 2B). The intermediate form would lack the DR_L_ but still possess the TMR repeats capable of forming a second t-loop, recombine and excise in the TMR of the DR_R_. This first possibility could also explain the presence of a single integrated DR if only one t-loop excision is made. Another possible excision mechanism could be a t-loop formation, recombination and t-loop excision of the DR_L_ in the DR_R_ directly (Figure 1B). The two possible mechanisms result into a circular viral genome with a single DR that has one *pac*1 and one *pac*2 sequence (Figure 2C). Because ciHHV-6A/B can be excised from chromosomes and form viral episomes, this suggests that integration is possibly a mode of latency for these viruses. 

## 6. How Does HHV-6A/B Integration Occurs?

**Figure 3:**
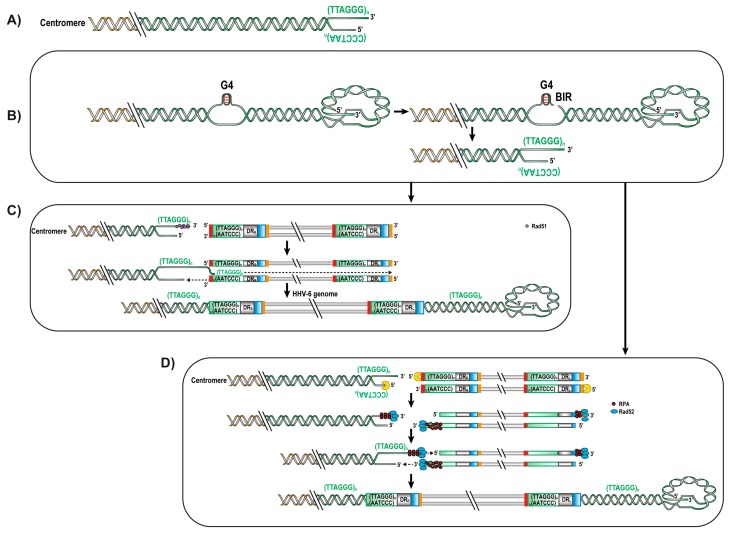
Possible mechanisms for HHV-6A/B integration. Schematic representation of HHV-6A/B chromosomal integration process. (**A**) Unfolding of the chromosome t-loop and invasion by the telomeric 3′ overhang into HHV-6A/B’s DR. This mechanism is unlikely to occur since all ciHHV-6A/B reported so far have lost most telomeric repeats. (**B**) Break induced replication (BIR) repair mechanism caused by G quadruplexes (G4) structure (or other blockage) in the lagging strand. (**C**) The free 3′ strand is rescued by Rad51 protein that searches for proximal homologous sequences. If a HHV-6A/B genome is close to proximity, Rad51 invades the viral TMR, displacing one strand of the HHV-6A/B genome to allow the synthesis of the complementary strand. Upon cell divisions, the DR_L_ would lose *pac1* due to end replication problem and the impTMR would serve as telomeric template to elongate telomeres at the end of the genome. (**D**) Single stranded annealing (SSA) repair mechanism. Upon virus entry in the cell, DNA damage response is triggered, at the same time a break caused by a stalled replication fork at the human telomeres activate SSA. SSA activation leads to resection of both the viral and human DNA in a 5′ to 3′ direction to create complementary sequences. Meanwhile, the 3′ strands of both genomes are protected by the replication protein A (RPA). Rasd52 binds the RPA and searched for pairing in which *pac2* will be lost. Annealed sequences then lead to the copying of the viral genome. Genomes are not drawn to scale.

Since HHV-6A/B both have TMRs and integrate into telomeres, it is presumed that these sequences play a role in viral integration. This hypothesis was confirmed by Wallasheck et al. that demonstrated that TMRs are important for HHV-6A/B telomeric integration [75]. However, the underlying mechanism of how HHV-6A/B integrates into chromosomes remains unknown. Sequence analysis of integrated HHV-6A/B genomes indicates that viral integration occurs very close to the subtelomeric region with few telomeric repeats between the viral genome and the chromosome [21,76]. Considering this, invasion of the viral TMR by the chromosome single-stranded 3′ extremity appears unlikely (Figure 3A). 

### Homologous Recombination

Homologous recombination is a mechanism used by all cells in the human body to repair double stranded DNA breaks. Under normal conditions, cells experience an average of 50 breaks per cell cycle [77]. Interestingly, HR is also used by telomerase-deficient cells like certain cancer cells to extend their telomeres to become immortalized. This mechanism is called Alternative Lengthening of Telomeres (ALT) [78,79]. As mentioned, telomeres must be able to prevent DNA damage responses to protect chromosomes. When a DNA break occurs, DNA damage response proteins such as H2AX, 53BP1, chK2, BRCA1 and p53 are recruited at the break [80]. ATM is then activated and recruits the MRN complex (Mre11, Rad50, NBS1) to the damaged sites. The accumulation of DNA damage response proteins leads to the recruitment of effector proteins such as Rad51, responsible for the 3′ strand invasion of a homologous region to repair the break. 

One possible scenario that could lead to HHV-6A/B integration is break-induced replication (BIR). During DNA replication, the cellular DNA polymerases can be stalled due to a variety of reasons including G quadruplex structures (G4). G4 are planar quaternary guanine rich secondary structures that can form after the unwinding of the DNA helix by DNA helicases [81,82,83]. G4 can block the replication fork and causes instability leading to DNA breaks that are generated to help repair this obstruction (Figure 3B). During replication of the telomere, it is the lagging strand-containing the TTAGGG motifs that can adopt G4 topologies and cause stalling of the replication fork. A break in the lagging strand generates a 3′ overhang that in association with Rad51 will search for homologies in nearby DNA. If a HHV-6A/B genome is in proximity, invasion can take place in the viral TMR. Following invasion, one strand of the HHV-6A/B genome gets displaced to allow the synthesis of the complementary strand (Figure 3C). The structures of most integrated HHV-6A/B genomes suggest that invasion by the cellular telomeric DNA occurs in the DR_R_’s TMR [21,76,84]. In such orientation, the DR_L_ would be at the end of the chromosome. Upon cell division and DNA replication, there would be loss of *pac*1 (due to the shortening of telomeres upon cell division) and the adjacent impTMR could serve as a template for the telomerase complex and telomere elongation, as previously reported [21,74]. On rare occasions, a single DR (without the rest of the genome) is detected suggesting that invasion can also occur in DR_L_’s TMR. Alternatively, such a structure could have arisen through partial excision of the viral genome as previously reported [21]. Interestingly, a recent report indicates that BRACO-19, a compound that binds and stabilizes G quadruplexes, affected the ability of HHV-6A to integrate into host chromosomes [62]. The exact mechanisms of BIR remain unknown but since BIR is a naturally-occurring mechanism, the presence of viral proteins may not be necessary except to bring the viral genome near the telomeres and/or DNA breaks. 

A second mechanism that could lead to viral integration is the single stranded annealing (SSA) repair mechanism, an HR pathway that uses homologous ends to repair the breaks (Figure 3D) [85]. Upon entry of the virus into a cell, the presence of a viral linear genome is likely to trigger a DNA damage response. Activation of SSA would recruit MRN to the viral DNA, resecting the viral DNA at both extremities in a 5′ to 3′ direction. In doing so, the single-stranded TMR at the 3′ extremity of DR_R_ would become complimentary to the single-stranded DNA generated following the break at the stalled the replication fork (Figure 3B). The replication protein A (RPA) binds the single stranded sequences to protect them, Rad52 joins RPA and the complex searches for complimentary pairing. During the pairing process, the *pac*2 site is lost. Once the two strands are annealed, the viral genome is then copied. Integration through SSA would not allow for integration to occur via DR_L_ as the single-stranded 3′ end would not complement the single-stranded DNA generated following the break at the stalled the replication fork. 

## 7. Conclusions

Though reported 25 years ago, the study of HHV-6A/B integration remains understudied. With the development of HHV-6A/B integration systems [86] and genome editing technologies, scientists should be able to tease out in detail the mechanisms leading to viral integration. Considering a prevalence of approximately 1%, pinpointing the association between iciHHV-6A/B and diseases is not trivial. This will require considerable effort, funding and access to large biobanks linked to detailed medical records. Scientists will likely need to share and pool their data to reach meaningful conclusions, especially for less-prevalent diseases. Furthermore, considering that appearance of a disease is likely to be influenced by the chromosome carrying the integrated HHV-6A/B as well of the sex of the individual, these variables should be taken into consideration during data analyses.
